# Influence of Hot-Etching Surface Treatment on Zirconia/Resin Shear Bond Strength

**DOI:** 10.3390/ma8125409

**Published:** 2015-11-30

**Authors:** Pin Lv, Xin Yang, Ting Jiang

**Affiliations:** Department of Prosthodontics, Peking University School and Hospital of Stomatology, Beijing 100081, China; lvpinyang@bjmu.edu.cn (P.L.); yangxinlv@bjmu.edu.cn (X.Y.)

**Keywords:** hot-etching, shear bond strength, zirconia

## Abstract

This study was designed to evaluate the effect of hot-etching surface treatment on the shear bond strength between zirconia ceramics and two commercial resin cements. Ceramic cylinders (120 units; length: 2.5 mm; diameter: 4.7 mm) were randomly divided into 12 groups (*n* = 10) according to different surface treatments (blank control; airborne-particle-abrasion; hot-etching) and different resin cements (Panavia F2.0; Superbond C and B) and whether or not a thermal cycling fatigue test (5°–55° for 5000 cycles) was performed. Flat enamel surfaces, mounted in acrylic resin, were bonded to the zirconia discs (diameter: 4.7 mm). All specimens were subjected to shear bond strength testing using a universal testing machine with a crosshead speed of 1 mm/min. All data were statistically analyzed using one-way analysis of variance and multiple-comparison least significant difference tests (α = 0.05). Hot-etching treatment produced higher bond strengths than the other treatment with both resin cements. The shear bond strength of all groups significantly decreased after the thermal cycling test; except for the hot-etching group that was cemented with Panavia F2.0 (*p* < 0.05). Surface treatment of zirconia with hot-etching solution enhanced the surface roughness and bond strength between the zirconia and the resin cement.

## 1. Introduction

High-strength materials such as zirconia can offer improved fracture resistance and longer-term viability [1,2]. However, the inertness of zirconia has made resin-to-zirconia bonding challenging [3]. The success of resin bonding relies on mechanical bonding through micromechanical interlocking from surface roughening, and chemical bonding between ceramic and cement [4,5]. Traditional methods of mechanical and adhesive bonding that are used on silica-based ceramics are not applicable with zirconia.

There has been extensive research concerning the adhesion between zirconia and resin cements [6]. Particle-air-abrasion using Al_2_O_3_ particles and different air pressures can be used to roughen the surface of zirconia [7,8]. Tribochemical silica coating in dental laboratories has also been suggested as an effective surface treatment [9,10]. However, several studies have demonstrated that these procedures can create surface microcracks that can decrease the strength and the apparent fracture toughness [11,12]. Recently, a novel surface roughening technique called selective infiltration etching (SIE) has been explored for zirconia [13]. Our previous study [14] revealed an improvement in bond strength using modified SIE technology but it was not up to clinical standards. There is currently no consensus regarding the best surface treatment for achieving optimum bond strength without altering the strength properties of zirconia.

Metals and zirconia have some similarities. For this reason, hot-etching technology, which was initially used with nickel chromium-alloy Maryland bridge wings [15], was explored with zirconia. Hot-etching technology provided a corrosion-controlled process. The grain structure on the zirconia surface was enlarged and the less-arranged, high-energy peripheral atoms were removed through the process. Casucci [16] proved that hot-etching could form the ideal surface roughness of zirconia. However, El-Korashy [17] noted that using a hot-etching surface treatment on zirconia formed unfavorable deep grooves which decreased, rather than increased, the bond strength to the resin cement. The inconsistency of these results may stem from the use of different hot-etching methods. The feasibility of hot-etching of zirconia clearly warrants further study.

The aim of this *in vitro* study was to actualize hot-etching technology with an effective and simple application using a reaction kettle, and to investigate changes in the surface morphology and bond strength between zirconia and resin cements after the etching procedure. The null hypotheses of this study were that the use of the surface hot-etching treatment would not influence the surface roughness of zirconia and would not affect the adhesion between zirconia and the resin cements.

## 2. Materials and Methods

### 2.1. Preparation of Zirconia Specimens

A total of 120 specimens of zirconia were compacted and sintered into dense disc specimens (thickness: 2.5 mm; diameter: 4.7 mm) from yttria-stabilized zirconia powder (97% zirconium, 3% Y_2_O_3_; Tosho, Tokyo, Japan) by the Biomaterials Department of Tsinghua University (sample density: 6.05 g·cm^−3^; flexural strength: 1300 MPa; Wechsler hardness (Hv): 12.5 GPa). The specimens were sequentially surface polished with SiC abrasive papers from grit #180, 240, 480, 600, 1000, 1200 to 2000 (P01–P12; Penda, Shanghai, China) under flowing water, rinsed with tap water for 1 min, ultrasonically cleaned in a deionized water bath for 30 min and gently air-dried.

Specimens were divided into three groups (each of *n* = 40) according to the different surface treatments performed:

- Group 1: blank control;
- Group 2: airborne-particle-abrasion with 50 μm Al_2_O_3_ particles applied for 10 s at 0.25 MPa of air pressure; and
- Group 3: hot-etching treatment for 1 h. Specimens were placed in a reaction kettle (KH-200; Zhongkaiya, Yancheng, China) that was filled with hot-etching solution (800 mL of methanol, 200 mL of 37% HCl and 2 g of ferric chloride) [18]. The kettle was heated to a constant temperature in a 100 °C water bath with magnetic stirring (MS-400; Bante, Shanghai, China). A rotor was placed in the reaction kettle and rotated at 40 rev/s by the magnetic stirring apparatus.

After these treatments, the specimens were rinsed with tap water for 1 min, ultrasonically cleaned in a deionized water bath for 30 min, and gently air-dried.

### 2.2. Evaluation by Scanning Electron Microscopy (SEM)

Each zirconia disc was rinsed with 96% ethanol and air-dried, mounted on metallic stubs, gold sputter-coated for 80 s (E-104S; Hitachi, Tokyo, Japan) and evaluated under a SEM (S-4800; Hitachi, Tokyo, Japan) at 25,000× magnification to assess the changes in surface topography.

### 2.3. Evaluation by Atomic Force Microscopy (AFM)

Each zirconia disc was imaged using Atomic Force Microscopy (AFM) (SPA300HV; Seiko, Tokyo, Japan). Five random 5 μm × 5 μm spots on the surface of each disc were imaged at a probe speed of 1 Hz (OMCL-TR400PSA; Olympus, Tokyo, Japan) at 256 × 256 resolution. SPI4000 software (NSK, Tokyo, Japan) was used for analyzing the image and the surface roughness. The average surface roughness (Ra) values and standard deviation for each specimen were calculated.

### 2.4. X-ray Diffraction (XRD)

XRD analysis (Max B; Shimadzu, Tokyo, Japan) using Cu Kα radiation was conducted to examine the influence of the different surface treatments on the phase composition and transformation of the zirconia. Zirconia surfaces were scanned with Cu Kα X-rays over an angular range of 2θ = 15°–75° with a step size of 0.026° and a 5 s step interval. The maximum peak intensities of the monoclinic *m* ($11\bar{1}$), *m* (111) and tetragonal *t* (111) phases were used to determine the extent of phase transformations after the different surface treatments. The relative amount of the monoclinic phase (Xm) was calculated according to the formulas proposed by Garvie and Nicholson [19].

### 2.5. Preparation of an Isolated Tooth

The Ethics Committee of the Hospital of Stomatology, Peking University (PKUSSIRB-201520021) granted permission to use isolated teeth.

A total of 120 human middle incisors that were free of caries, fluorosis, cracks, or previous endodontic treatments were selected from incisors recently extracted for heavily reduced periodontal support at the Hospital of Stomatology, Peking University. The teeth were stored in 0.5% chloramine solution at 4 °C and external debris was removed using a hand scaler. The root part of each tooth was sectioned 1 mm below the cement-enamel junction with a diamond burr (TR11, Mani, Japan) to obtain the crown. Each crown was embedded in self-curing resin in a Teflon mold to form a cylindrical specimen (diameter: 20 mm; length: 40 mm). The labial surface of the crown was exposed at the top of the cylinder. After solidification of the self-curing resin, the labial surface of the crown was polished with SiC abrasive paper (gradually from grit #180, 240, 360, 600, 1000 to 2000) under flowing water to achieve a uniform 5 mm × 5 mm enamel surface. The isolated tooth specimens were discarded when the area of the exposed enamel was less than 5 mm × 5 mm or if a chalky color surface was not observed after etching by 35% phosphoric acid in the step described below.

### 2.6. Cementing Procedure and Thermocycling Test

The zirconia specimens from the three different treatment groups were further subdivided according to the two different resin cements (Superbond C and B 4-META-type self-polymerized resin cement, Sun Medical, Moriyama City, Japan; Panavia F2.0 MPD-based resin cement, Kuraray Noritake, Japan) and whether or not the thermocycling fatigue testing was performed. Each subgroup contained 10 specimens.

The treated surfaces of the zirconia were cemented to the enamel surfaces according to the manufacturer’s instructions. In the Superbond C and B subgroup, the enamel surfaces were etched with a layer of red Activator Superbond C & B for 30 s, rinsed and then dried. The Superbond C and B monomer, catalyst and polymer were mixed at a ratio of 4:1:1 and the mixture was then applied to the surface of each zirconia surface. In the Panavia F2.0 subgroup, the enamel surfaces were etched with a layer of phosphoric acid (Etch 35 Gel; Heraeus, Hanau, Germany) for 30 s, rinsed for 15 s and air-dried. ED Primers A and B were combined and painted on the enamel surface for 30 s and air-dried. The combined A and B paste was applied to the surface of the zirconia.

The zirconia specimens were cemented on the surface of the enamels and then compressed at a constant load of 10 N for 20 s; excess cement was then wiped off. The specimens of the Panavia F2.0 group were light-cured for 40 s, then all of the specimens were stored in distilled water for 24 h.

Half of each group was subjected to a thermocycling test (TC-501-F Thermocycling Device, Suzhou, China) in deionized water for 5000 cycles between 5 and 55 °C. The dwell time at each temperature was 30 s and the transfer time was 2 s (ISO 10477-1996) [20].

### 2.7. Shear Bond Strength Test and Fracture Mode Examination

Each specimen was loaded onto a universal test machine (Instron; Norwood, MA, USA) equipped with a 1000 kg load cell. The shear bond strength test was performed according to the guidelines specified in the ISO/TS 11405-2003 [21]. The shear force was applied parallel to the interface of the bonding surfaces at a speed of 1 mm/min until bonding failed. The shear force was recorded automatically at the point of failure, with the value taken as indicating the shear bond strength (MPa).

After debonding, the fractured interfaces of the specimens were examined with a stereomicroscope (OCS 912042; Olympus, Tokyo, Japan) at 10× magnification to determine the debonding modes as follows: ZR, failure between the zirconia surface and resin cement; ER, failure between the enamel and resin cement; MM, mixed mode; and CF, cohesive fracture inside the resin cement.

### 2.8. Statistical Analysis

One-way analysis of variance (ANOVA) in combined with least significant difference (LSD) tests performed with the IBM SPSS Statistics (version 22.0, IBM SPSS, Chicago, IL, USA) were used to analyze differences in surface roughness and the shear bond strength values among groups (α = 0.05), after the normal distribution and homogeneity of variance were checked using the Shapiro-Wilk test and Levene tests, respectively.

## 3. Results

**Figure 1:**
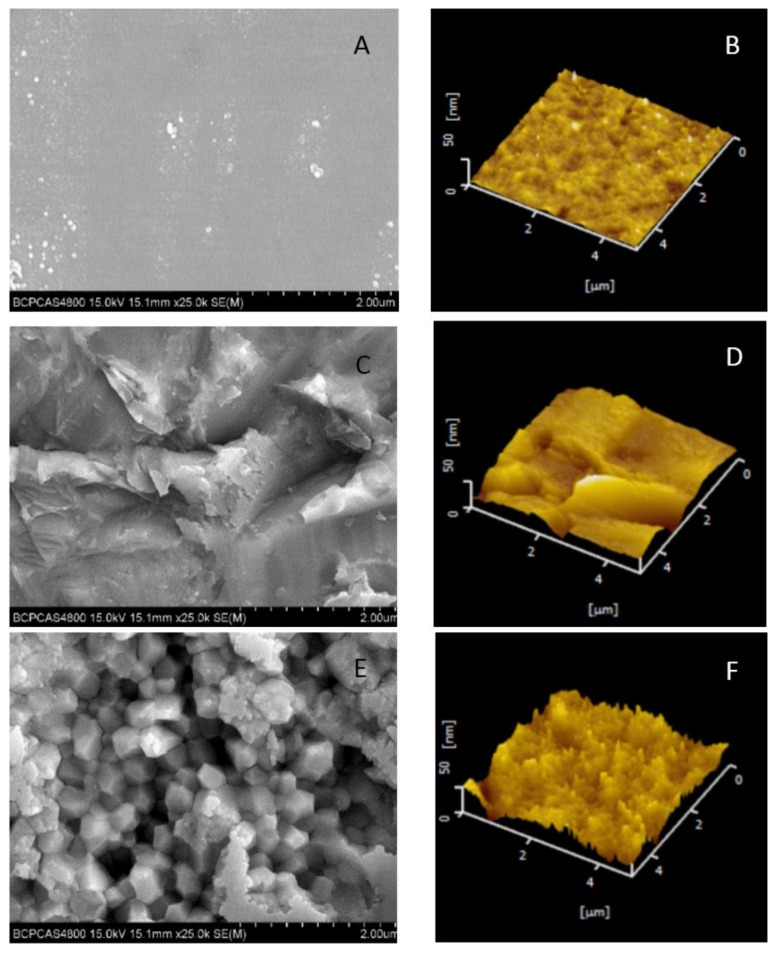
Scanning electron microscopy photomicrographs and atomic force microscopy images of zirconia ceramic discs after different surface treatments. (**A**,**B**) blank control; (**C**,**D**) airborne-particle-abrasion; and (**E**,**F**) hot-etching treatment.

Representative SEM and AFM images of each group were shown in Figure 1. The mean values and standard deviations of the average surface roughness (Ra) of the zirconia surfaces were extrapolated from these digital images (Table 1). The hot-etching group had significantly higher Ra values compared to the control group (*p* < 0.01). No significant difference was detected between the airborne-particle-abrasion and hot-etching groups (*p* > 0.05). The shear bond strength of each group was listed in Table 2. The hot-etching group had significantly higher bond strength than the control and airborne-particle-abrasion groups not only in the immediate test but also after thermal cycling (*p* < 0.01). The bond strengths were significantly lower after the thermal cycling test in all groups (*p* < 0.05) except for the hot-etching group that was cemented with Panavia F2.0 (*p* = 0.08).

**Table 1:** Surface roughness (Ra, nm) and X_M_ of zirconia with different surface treatments.

Surface Treantment	Surface Roughness	X_M_ (%)
no treatemnt	1.31 ± 0.96 ^a^	9.1
airborne particle abrasion	6.64 ± 2.07 ^b^	21.9
hot-etching	6.41 ± 0.49 ^b^	7.4

^a,b^ indicated significant differences (*p* < 0.05).

**Table 2:** The shear bond strength (MPa) of zirconia with different surface treatments (Mean ± standard deviation).

Surface Treatments	Resin Cements			
	Superbond C & B		Panavia F2.0	
	Immediate Bond Strengths	Bond Strengths after Thermal Cycling	Immediate Bond Strengths	Bond Strengths after Thermal Cycling
no treatment	23.37 ± 3.99 _a_^A^_α_	9.61 ± 3.34 _a_^A^_β_	18.12 ± 4.73 _b_^A^_α_	12.91 ± 3.33 _a_^A^_β_
airborne particle abrasion	26.06 ± 3.14 _a_^A^_α_	15.8 ± 4.25 _a_^B^_β_	23.2 ± 5.47 _a_^B^_α_	16.97 ± 2.9 _a_^A^_β_
hot-etching	34.43 ± 1.84 _a_^B^_α_	29.25 ± 4.46 _a_^C^_β_	32.1 ± 7.73 _a_^C^_α_	28.13 ± 6.53 _a_^B^_α_

Significant differences are indicated by different subscripted lower case letters (_a, b_) (within a row for the same shear bond test method), or by different superscripted upper case letters (^A,B,C^) (within a column), or by different subscripted lower case letters (_α_, _β_)(within a row comparing immediate bond strength and bond strength after thermal cycling for the same resin cement) (α = 0.05).

**Figure 2:**
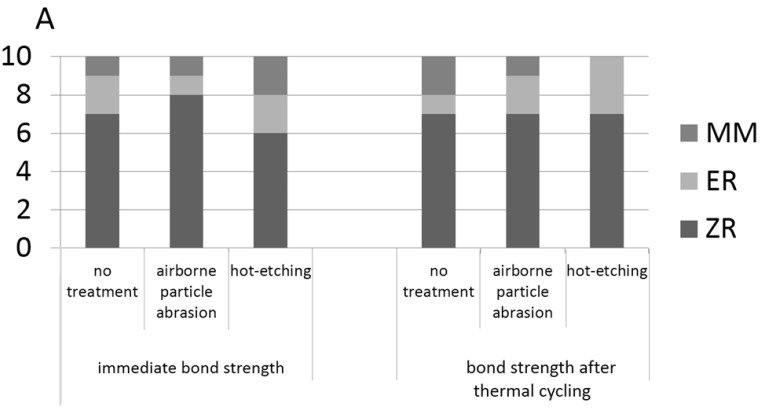

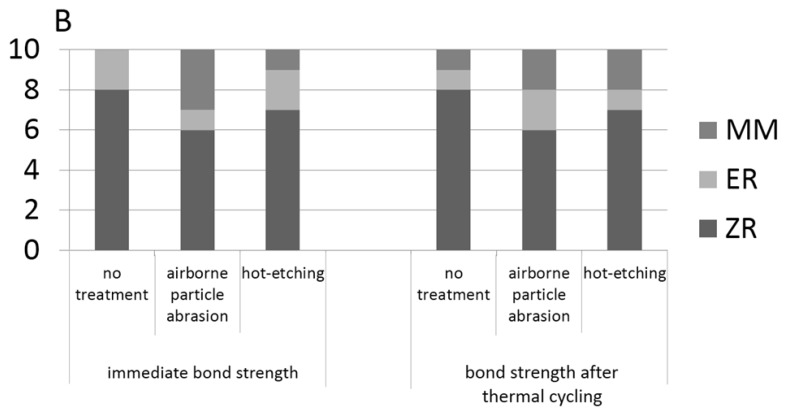
Percentage of different failure modes in each group. (**A**) Bonded with Superbond resin cement; and (**B**) bonded with Panavia F2.0.

**Figure 3:**
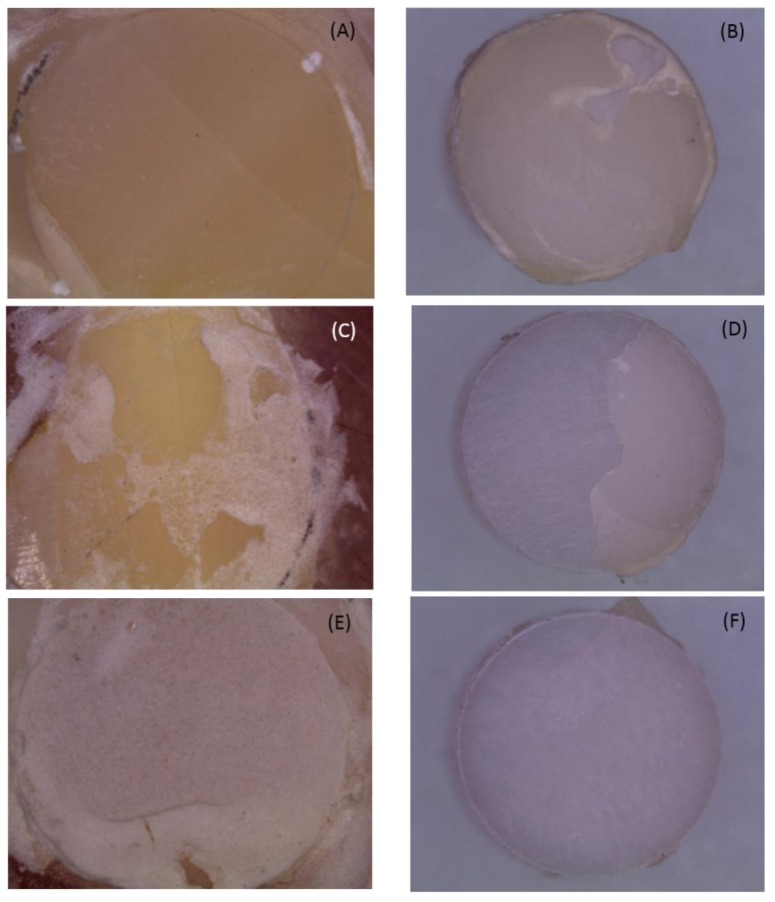
Representative stereomicroscope photomicrographs of the fracture surfaces of enamel and zirconia. (**A**,**B**) ER (adhesive fracture between enamel and resin); (**C**,**D**) MM (mixed fracture mode); and (**E**,**F**) ZR (adhesive fracture between zirconia and resin).

The distribution of the different failure modes of each group was shown in Figure 2. Cohesive failure in the resin cement was never observed and most of the failure modes were ZR. Figure 3 showed stereomicroscope photomicrographs of the fracture surfaces of the enamel and zirconia.

**Figure 4:**
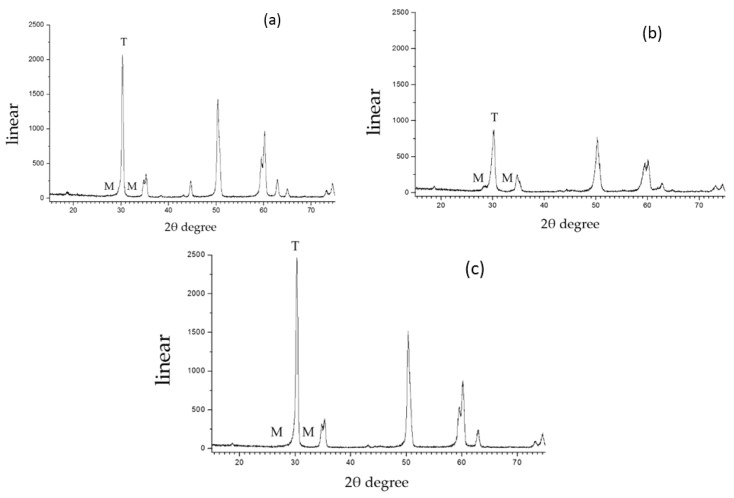
X-ray diffraction (XRD) analyses of each group: (**a**) blank control; (**b**) airborne-particle-abrasion; and (**c**) hot-etching. *T* indicates the tetragonal zirconia phase and *M* indicates the monoclinic zirconia phase.

Figure 4 shows the representative XRD patterns of the zirconia; X_M_ was calculated from these patterns. The amount of monoclinic phase was negligible in the control and hot-etching groups, and the airborne-particle-abrasion group had a higher relative amount of the monoclinic zirconia phase.

## 4. Discussion

### 4.1. Hot-Etching Technology

The use of hot-etching treatment on zirconia has been recently studied [22]. The action of the hot acid solution is basically a corrosion-controlled process [18]. It is proposed that the solution chemically etches the grain structure on the zirconia surface, enlarging the grain boundaries through the preferential removal of less-ordered, high-energy peripheral atoms. The acid solution was comprised of methanol, concentrated hydrochloric acid, and ferric chloride. Methanol is the solvent, the concentrated hydrochloric acid provides adequate hydrogen ions, while the ferric chloride is the primary corrosive agent. The etching rate depends on solution movement over the ceramic surface; the solution was heated to aid the dissolution process.

In the present study, a simple kind of reaction kettle was used to carry out the hot-etching process. The reaction kettle was of a type widely used in the chemical field for acid etching. The inner tank of the kettle was made of polytetrafluoroethylene (PTFE), which is an extremely stable material, and it was covered with a shell made of stainless steel. The test temperature (100 °C) was suitable for conditioning the substrate in a water bath application. The boiling point of methanol is 64.7 °C, so a pressure-tight reaction kettle was needed to heat the solution to the reaction temperature. The etching rate inside the kettle was accelerated by using a rotor that was activated by the magnetic agitator.

### 4.2. Surface Roughness and XRD Analysis

Strong resin bonding relies on micromechanical interlocking and adhesive chemical bonding to the ceramic surface, requiring surface roughening for mechanical bonding and surface activation for chemical adhesion [23]. The present study demonstrated that the hot-etching treatment significantly increased the average surface roughness of the zirconia (*p* < 0.01); these results are in agreement with Casucci *et al.* [24]. The etching effect of the hot solution was revealed in the SEM and AFM photomicrographs (Figure 1), which showed cellular concavities on the zirconia surfaces. This corroborated the measured roughness values that were significantly higher than those of the blank control group (*p* < 0.01).

XRD analysis revealed that the airborne-particle-abrasion group contained a higher relative amount of the monoclinic zirconia phase, in agreement with other studies [25,26]. The monoclinic phase may contain microcracks and flaws that could compromise the long-term stability and reliability of zirconia [27], which could explain the significantly lower bond strength for the airborne-particle abrasion group relative to the hot-etching group, despite both groups having approximately the same surface roughnesses (*p* < 0.05).

### 4.3. Shear Bond Strength Test

The present study demonstrated that the hot-etching treatment significantly enhanced the shear bond strength of resin cement to zirconia when compared with no treatment and airborne-particle-abrasion treatment for both immediate testing and testing after thermal cycling (*p* < 0.05). The cellular structure etched by the acid solution was of a form favorable for micromechanical interlocking with the resin cement as well as to the enamel after a three-step etch-and-rinse process. This result contradicts those of El-Korashy [17] who concluded that deep grooves created by a hot-etching treatment would decrease the bond strength to the composite cement. When compared with our previous study of the modified SIE treatment, the effect of hot-etching treatment gave improved shear bond strength between the zirconia and the resin cement.

After thermal cycling, the shear bond strength significantly decreased for all of the groups except for the hot-etching group bonded with Panavia F2.0 (*p* < 0.05). This decrease might relate to the different coefficients of thermal expansion of the bonded substrates. Since Panavia F2.0 contains the phosphate ester monomer MDP (methacryloyloxydecyl dihydrogen phosphate), some reaction may have occurred between the adhesive monomer and the zirconium oxide layer coated on the zirconia surface [28]. Combined with the surface roughness achieved by the hot-etching treatment, the decrease of bond strength caused by thermal cycling could be reduced.

Superbond resin cement containing 4-META, and Panavia F2.0 containing 10-MDP, are commonly used in the clinic. In previous studies, these two cements were recommended for bonding to zirconia having different surface treatments [29,30,31]. Table 1 reveals that under the same treatment, the group bonding with Superbond cement reached a higher immediate bond strength than the group bonding with Panavia. This is in accordance with the study of Per Dérand [32]. However, in the present study, except for the immediate bond strength of the control group, there was no significant difference between them (*p* > 0.05).

The failure mode observed was mostly failure between the ceramic surface and the resin cement. The shear bond strength could reach its ultimate value under this failure condition.

The present study indicated that the use of the hot etching technology as a pre-treatment of zirconia significantly enhanced the adhesion between zirconia and resin cement (*p* < 0.05). This treatment is an alternative treatment to airborne-particle-abrasion and avoids micro-crack formation and phase transitions that are detrimental to the longevity of the ceramic restoration. However, the hot-etching treatment still requires further study before being used as a step in the processing of zirconia restorations: control of the hot-etching area, alteration of cytotoxicity and biaxial flexural strength after hot-etching are all issues that need attention. The limitation of this study was that only one hot-etching reaction time was used, the optimal reaction time and other technology parameters need to be explored in future work: only one zirconia quality, combined with two resin composite cements, were tested; the universal property of this hot-etching method needs to be researched.

## 5. Conclusions

Within the limitations of this laboratory study, the following conclusions are drawn:

1. Hot-etching technology could be performed in a simple reaction kettle;
2. Hot-etching treatment produced significantly higher shear bond strength between zirconia and resin cement compared to no treatment and airborne-particle-abrasion treatment (*p* < 0.05); and
3. Hot-etching treatment could provide a rougher zirconia surface while avoiding the detrimental tetragonal-to-monoclinic surface phase transition.
